# Subinhibitory Antibiotic Concentrations Enhance Biofilm Formation of Clinical *Enterococcus faecalis* Isolates

**DOI:** 10.3390/antibiotics10070874

**Published:** 2021-07-19

**Authors:** Sara Bernardi, Annette Anderson, Guido Macchiarelli, Elmar Hellwig, Fabian Cieplik, Kirstin Vach, Ali Al-Ahmad

**Affiliations:** 1 Department of Life, Health and Environmental Sciences, University of L’Aquila, 67100 L’Aquila, Italy; guido.macchiarelli@univaq.it; 2 Centre of Microscopy, University of L’Aquila, 67100 L’Aquila, Italy; 3 Department of Operative Dentistry & Periodontology, Faculty of Medicine, University of Freiburg, 79106 Freiburg, Germany; annette.anderson@uniklinik-freiburg.de (A.A.); elmar.hellwig@uniklinik-freiburg.de (E.H.); ali.al-ahmad@uniklinik-freiburg.de (A.A.-A.); 4 Department of Conservative Dentistry and Periodontology, University Hospital Regensburg, 93053 Regensburg, Germany; fabian.cieplik@ukr.de; 5 Institute of Medical Biometry and Statistics, Faculty of Medicine, 79104 Freiburg, Germany; kv@imbi.uni-freiburg.de

**Keywords:** biofilms, subinhibitory antibiotic concentrations, *Enterococcus faecalis*

## Abstract

*Enterococcus faecalis* is a microorganism that can be found in the oral cavity, especially in secondary endodontic infections, with a prevalence ranging from 24–70%. The increase in the ability to form biofilms in the presence of subinhibitory antibiotic concentrations is a phenomenon that is observed for a wide variety of bacterial pathogens and is associated with increased resistance. In this study, therefore, six *E. faecalis* isolates from an endodontic environment and two control strains were exposed to subinhibitory concentrations of Penicillin G, Amoxicillin, Doxycycline, Fosfomycin, Tetracycline and Vancomycin and examined for their biofilm formation abilities. The minimum inhibitory concentration (MIC) was determined for all *E. faecalis* isolates. A culture of the isolate was mixed with a serial dilution series of the respective antibiotic, incubated overnight and the biofilm formation was analyzed using a microtiter plate assay. All isolates were able to form biofilms in the absence of an antibiotic. A significant increase in biofilm formation of up to more than 50% was found in the isolates exposed to subinhibitory concentrations of various antibiotics. Most isolates showed a significant increase in Fosfomycin (7/8), Doxycycline (6/8) and Tetracycline (6/8). Three endodontic isolates showed a significant increase in five of the antibiotics examined at the same time. On exposure to Vancomycin, three endodontic isolates and the two control strains showed an increase. The increase in the ability to form biofilms extended over a concentration range from 1/2 to 1/64 of the MIC concentration. Antibiotics may reach certain niches in the oral cavity at subinhibitory concentrations only. This can increase the biofilm formation by enterococci, and in turn lead to decreased susceptibility of these taxa to antibiotics.

## 1. Introduction

Microbial communities coexist in a matrix made of extracellular polymeric substance (EPS), resulting in the formation of a biofilm [1]. This type of microbial lifestyle provides the microorganisms with protection from physical (UV light and dehydration), chemical (antimicrobial molecules) and biological (immune cells) threats [2]. The EPS composition, made of polysaccharides and proteins and completed with open water channels, offers a hydrated shield to bacterial communities and influences the microorganisms’ phenotype [1,3,4]. Both abiotic and biotic surfaces can host biofilm development. The surfaces of the oral cavity provide an environment that hosts several types of biofilm, which can be involved in different infective diseases such as dental caries, periodontitis, halitosis, and endodontic infection [5,6,7,8]. While dental caries and halitosis represent conditions that may be prevented using oral hygiene strategies, advanced periodontitis, periimplantitis and endodontic infections are more complex conditions that also require preventive strategies, such as the development of bacteriostatic surfaces [9], the correct use of medicated mouthwashes [10], and the use of topical and systemic antibiotics [11]. The endodontic environment is morphologically difficult for microbicidal molecules and dental instruments to reach, and the presence of lateral canals or a particular variance of the apex can result in the failure of the endodontic therapy and the occurrence of secondary endodontic infection with apical bone suffering [12]. 

Since acute secondary endodontic infection can require the administration of systemic antibiotics [13], studies in the literature focused on the identification of the microbial communities’ species to direct clinicians towards the best compounds for treatment and to lower the risk for antibiotic resistance as much as possible [14]. When the antibiotic administration does not respect the prescribed scheme, the molecular concentration can be lower than the standard for the inhibitory purposes. This also applies to the local administration of disinfectant irrigants, due to the narrow space of the root canal. Moreover, such subinhibitory antibiotic concentrations may also occur after a standard antibiotic therapy in the surrounding area of infected root canals. Subinhibitory concentrations, i.e., concentrations of the antibiotic that are lower than the minimum inhibitory concentration (MIC) have been associated with several effects on the bacteria themselves, for example with a higher capacity for biofilm formation [15]. This in turn will result in a decreased susceptibility against antibiotics [16].

Recent studies reported how the subinhibitory concentrations of antibiotics can induce a stress to microorganisms living in biofilm communities, causing a selection of the more resistant bacteria at the genetic level and stimulating the biofilm formation [17,18]. In particular, Jin et al. reported how subinhibitory concentrations of a topical antibiotic, Mupirocin, not only induced the biofilm formation in *Staphylococcus aureus*, but also induced a changed expression of the *cidA* gene, which is involved in the cell death regulation in the biofilm environment [17]. Nagasawa et al. confirmed this phenomenon in another model using *Streptococcus mutans*, which is one of the main species involved in dental caries [18]. They found that subinhibitory concentrations of Bacitracin, that create a cell envelope stress, induced biofilm formation, influenced the balance of resistant and sensitive microorganisms and affected the genetic expression of the *atlA* and *rgp* genes that regulate the wall cell properties [18].

In endodontic infections, due to the peculiar anatomy of the canals and of the apex, the local administration of microbicidal irrigants such as NaOCl may not reach all the surfaces and thus leave microorganisms in the endodontic biofilm [19] that promote the occurrence of secondary infections. Secondary endodontic infections not only induce an activation of the cell-mediated immune system and are a potential source of focal disease [20] or of the Lemierre syndrome [21], but also affect the healing and the regeneration of the periapical bone, influencing the rate of tooth survival [22,23,24].

*Enteroccoccus faecalis* is a species frequently occurring in secondary endodontic infections (prevalence of 24–70%) [16]. Indeed, the microbial population of the primary and secondary infections differs, although both populations originate from the polymicrobial community of the oral cavity [25]. The microbial population of secondary endodontic infections include Gram-positive and facultative anaerobic bacterial species [26]. Therefore, understanding the effects of subinhibitory concentrations of antibiotics on *E. faecalis* and its capacity to form biofilm is fundamental to prevent the selection of resistant bacteria and for an appropriate use of these substances.

The aim of the present study was to evaluate the effects of different antibiotics at different subinhibitory concentrations on the biofilm formation of *E. faecalis*. For this purpose, six clinical *E. faecalis* isolates obtained from endodontic infections were exposed to different concentrations of six different antibiotics and the biofilm formation was analyzed for each concentration.

## 2. Materials and Methods

### 2.1. Isolates, Antibiotics and Inoculation

The evaluation of biofilm formation in the presence of subinhibitory antibiotic concentrations included six *E. faecalis* isolates from endodontic biofilms (12sp, 11sp, RGFR, 1R8, 1R1, 21sp) and two control species (ATCC 29,212 and laboratory strain T9).

The clinical isolates of *E. faecalis* were cultivated for 24 h on Columbia blood agar (CBA) plates at 37 °C in an aerobic atmosphere with 5% CO_2_. The bacteria were derived from long-term storage at −80 °C in brain heart infusion medium containing 15% (*v/v*) glycerol [27]. As previously described [28,29], the isolates were grown on blood agar plates for 24 h at 37 °C in an aerobic atmosphere with 5% CO_2_. The colonies were subsequently suspended in NaCl until the turbidity of the solution was similar to a 0.5 MacFarland scale (~10^8^ CFU/mL). The suspension was diluted 1:100 in TSB, which is equivalent to a concentration of 10^6^ CFU/mL. The inoculum was then further diluted in a 100-step series to reach a concentration 10^3^ CFU/mL. Subsequently, 100 µL were plated twice and incubated for 24 h. Finally, the CFU were counted and a bacterial suspension 6 × 10^6^ CFU/mL was obtained.

The antibiotic compounds tested were Amoxicillin sodium salt, Doxycycline hydrochloride, Fosfomycin calcium salt, Penicillin G sodium salt, Tetracycline hydrochloride and Vancomycin hydrochloride. All antibiotics were purchased from Sigma-Aldrich (Steinheim, Germany).

The MIC tests were performed according to the Clinical and Laboratory Standards Institute guidelines [30,31], as described earlier [32]. In detail, all antibiotics were serially diluted in TBS solution ranging from 100% to 0.78% (100% (undiluted), 50% (1:2), 25% (1:4), 12.5% (1:8), 6.25% (1:16), 3.12% (1:32), 1.56% (1:64), 0.78% (1:128)). The MIC was defined as the lowest concentration of antibiotic at which bacterial growth inhibition was visible [31]. All tests were carried out in duplicate.

### 2.2. Biofilm Formation Evaluation

As previously described in detail [28,29], the biofilm formation was evaluated with optical density parameters. After culturing the chosen isolates of *E. faecalis* and obtaining the dilutions as described above, the wells of polystyrene 96-well tissue-culture plates (Greiner bio-one, Frickenhausen, Germany) were filled with 100 µL of the diluted isolates and the diluted antibiotics (32 µg/mL–0.5 µg/mL) were added. The plates were incubated overnight [28,29] at 37 °C in an aerobic atmosphere with 5% CO_2_.

After discharging the culture medium, the 96-well-plates were washed three times using 300 μL phosphate buffered saline (PBS, Sigma-Aldrich Chemie GmbH), to remove the non-adherent bacteria. After air-drying, the adherent microorganisms that remained were stained with 0.1% crystal violet solution (Median Diagnostics GmbH, Dunningen, Switzerland) for 10 min. Excess dye was removed by rinsing the plates with distilled water. Afterwards, the plates were dried for 10 min at 60 °C and dye resolubilization was performed by adding 50 μL of absolute ethanol (99.9% *v/v*) (Merck Chemicals GmbH, Darmstadt, Germany) for analysis in each well. The optical density was measured using a Tecan Infinite-M200Plate-Reader (Tecan, Crailsheim, Germany) at a wavelength of 595 nm (OD595). All tests were conducted in quadruplicate and the mean values were determined.

### 2.3. Statistical Analysis

For descriptive analysis, mean values and standard deviations were calculated. Bar charts were used for graphical presentation of the results. Paired T-test was used to assess the concentrations’ increase by means of the OD values, comparing the antibiotic dilution exposure to growth control. T-tests were used for pairwise comparisons of the differences between biofilm formation in the presence of subinhibitory concentrations of the tested antibiotics within different isolate and MIC-subgroups. For the analyses, the statistics program STATA (StataCorp LT, College Station, TX, USA, Version 16.1) was used. The bar charts were realized using GraphPad Prism 9.1.1 (GraphPad Software, San Diego, CA, USA). 

## 3. Results

In the present study, the effects of subinhibitory concentrations of different antibiotics on biofilm formation of different clinical isolates and two control strains of *E. faecalis* were tested. The increase in OD value, representing an increased percentage of adhered cells, was the measure for the increase in biofilm formation. The results indicated that subinhibitory concentrations of each tested antibiotic were able to increase the biofilm formation of several clinical isolates of *E. faecalis*. At the same time, each of the isolates showed significantly increased biofilm formation with at least one and up to five of the applied antibiotics (Table 1).

**Figure 1:**
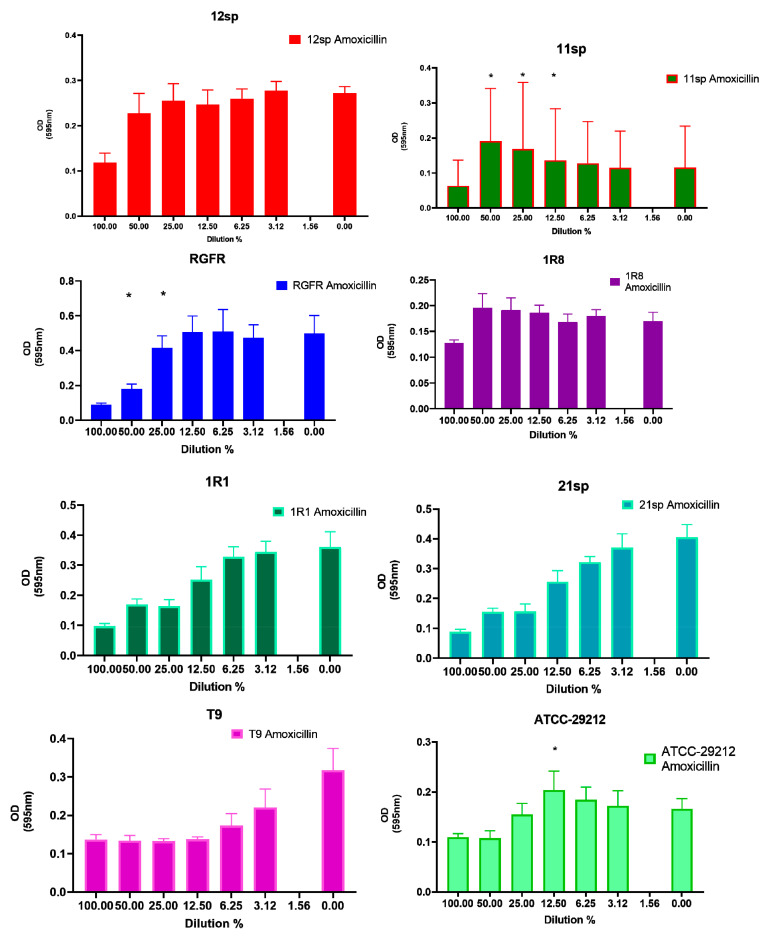
Graphical depiction of the biofilm formation (OD values) of each isolate exposed to subinhibitory concentrations of Amoxicillin. * indicates the significant values (*p* < 0.05), reported in Table 1. The % of the dilutions are shown on the X axes, and the OD values are shown on the Y axes.

Subinhibitory concentrations of Amoxicillin showed the least increase in biofilm formation, since only two tested isolates and one control isolate showed a significantly increased biofilm (*p* < 0.05) in the presence of 50%, 25% and 12.5% of the MIC (Figure 1).

**Figure 2:**
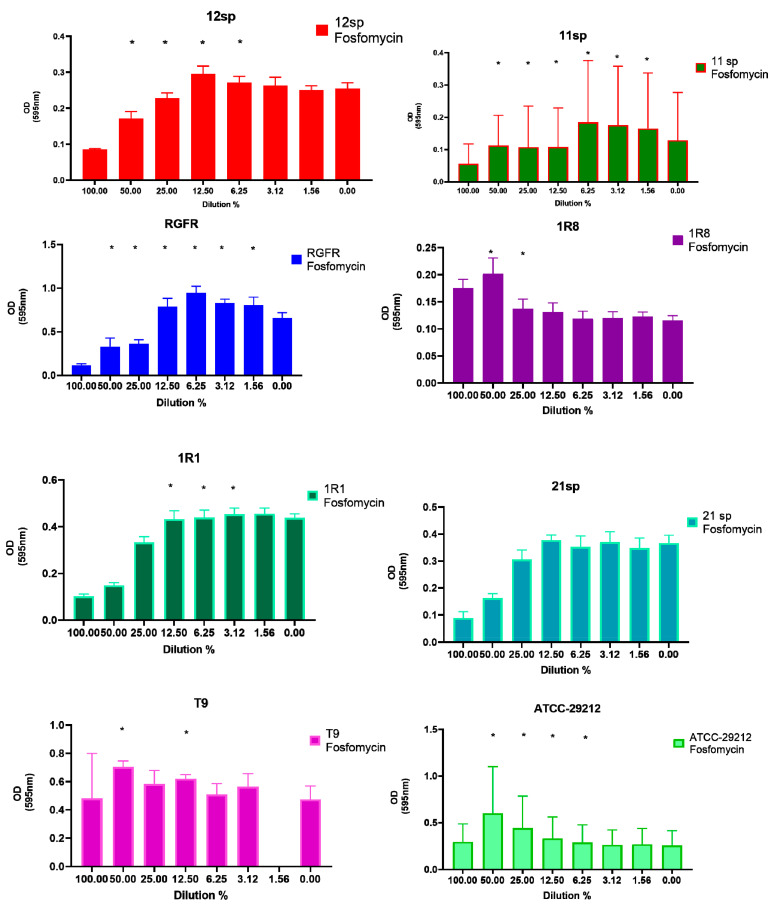
Graphical depiction of the biofilm formation (OD values) of each isolate exposed to subinhibitory concentrations of Fosfomycin. * indicates the significant values (*p* < 0.05), reported in Table 1. The % of the dilutions are shown on the X axes, and the OD values are shown on the Y axes.

**Figure 3:**
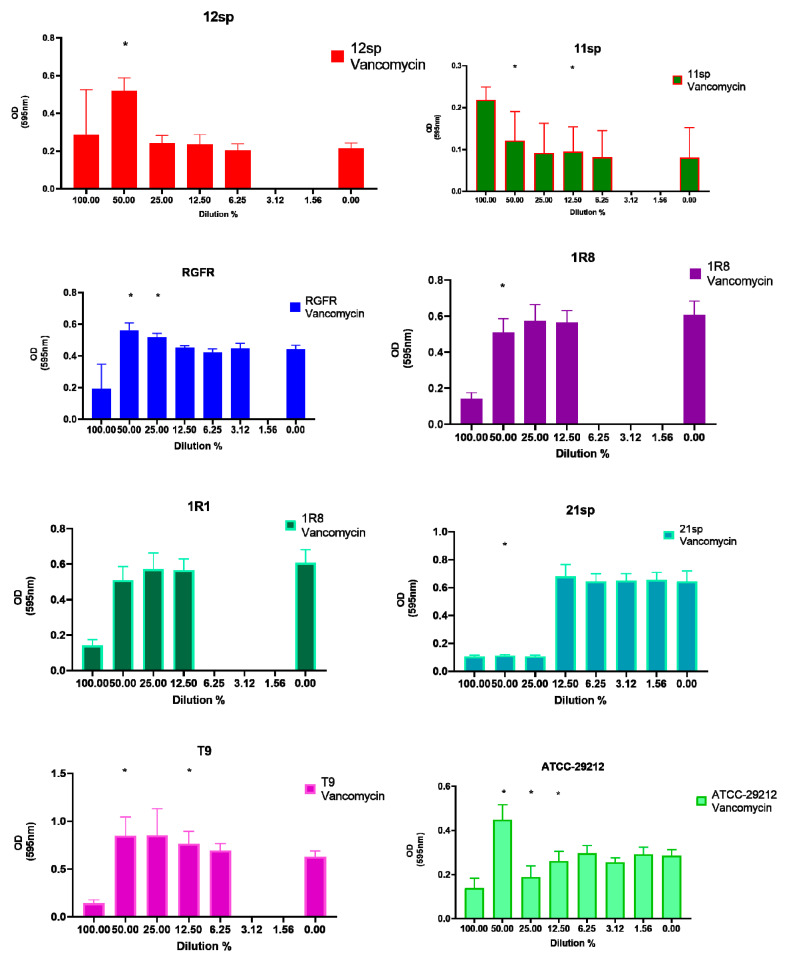
Graphical depiction of the biofilm formation (OD values) of each isolate exposed to subinhibitory concentrations of Vancomycin. Subinhibitory concentrations that induced a significant increase of OD values ranged from 50% to 12.5%. * indicates the significant values (*p* < 0.05), reported in Table 1. The % of the dilutions are shown on the X axes, and the OD values are shown on the Y axes.

**Figure 4:**
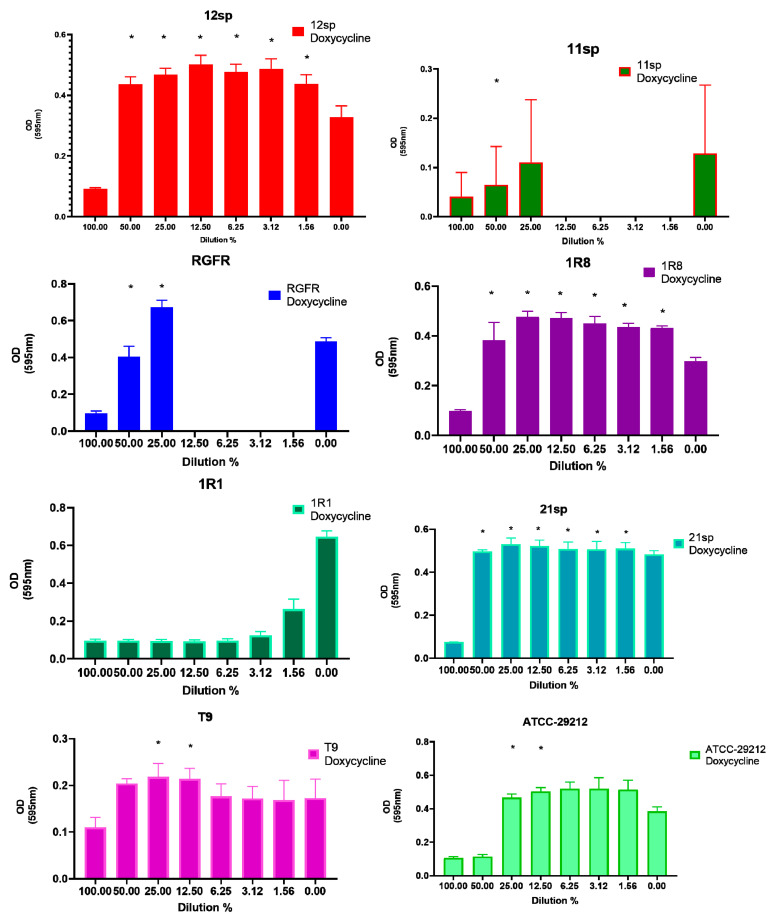
Graphical depiction of the biofilm formation (OD values) of each isolate exposed to subinhibitory concentrations of Doxycycline. * indicates the significant values (*p* < 0.05), reported in Table 1. The % of the dilutions are shown on the X axes, and the OD values are shown on the Y axes.

On the other hand, subinhibitory concentrations of Fosfomycin, Vancomycin and Doxycycline showed the greatest increase in biofilm formation: in all of the three tested antibiotics five clinical isolates and two control isolates (seven of the eight tested isolates in total) showed significantly (*p* < 0.05) increased capacity of biofilm formation in the presence of different subinhibitory concentrations (from 50% to 1.56% of MIC) (Figure 2, Figure 3 and Figure 4).

**Figure 5:**
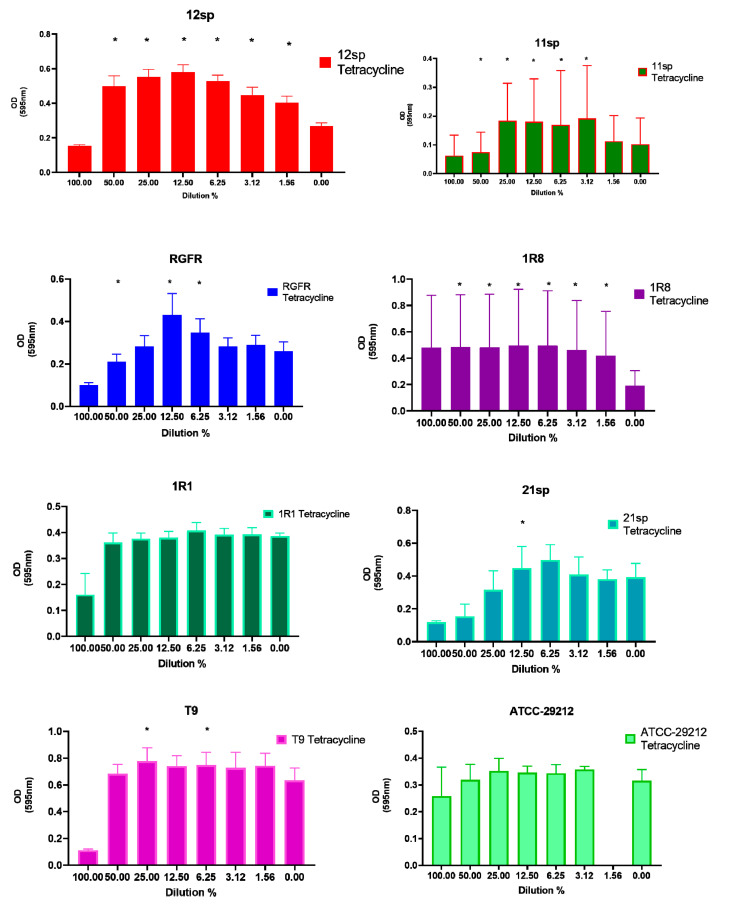
Graphical depiction of the biofilm formation (OD values) of each isolate exposed to subinhibitory concentrations of Tetracycline. * indicates the significant values (*p* < 0.05), reported in Table 1. The % of the dilutions are shown on the X axes, and the OD values are shown on the Y axes.

Data on the Tetracycline subinhibitory concentrations showed a significant (*p* < 0.05) increase of capacity of biofilm production in five clinical isolates and one control isolate in the presence of different subinhibitory concentrations (from 50% to 1.56% of MIC) (Figure 5). Regarding isolate 1R8, all antibiotic concentrations of tetracycline show a very large effect on the biofilm production and also almost all other tested antibiotics showed a high biofilm formation (already at MIC or ½ MIC) compared to growth without antibiotic.

**Figure 6:**
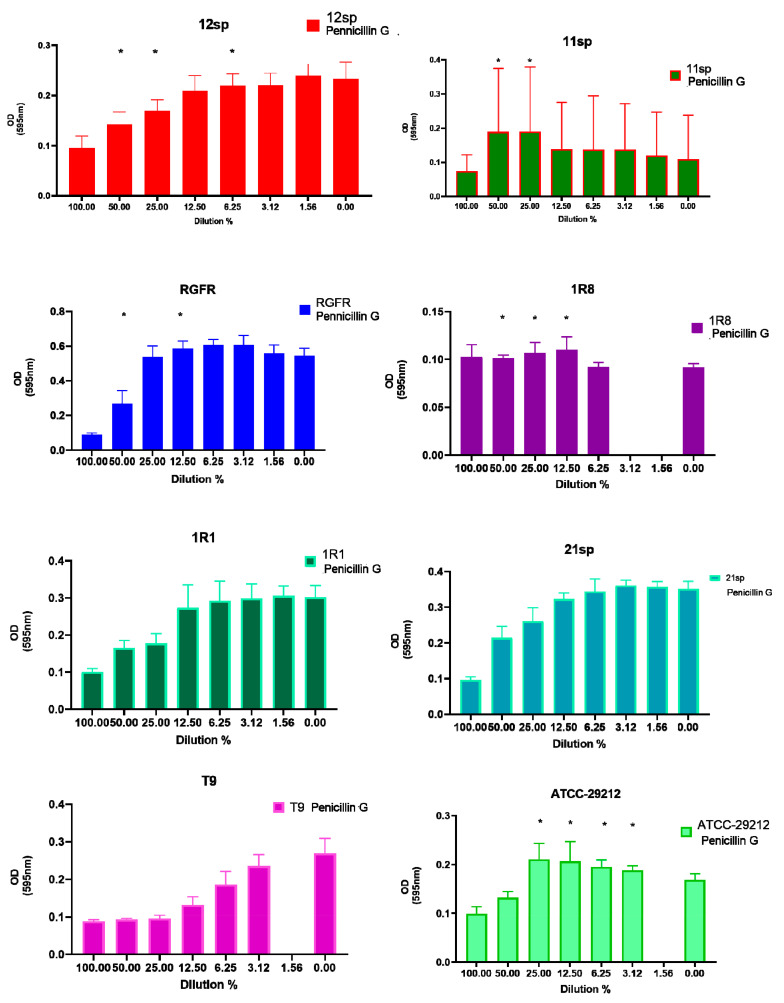
Graphical depiction of the biofilm formation (OD values) of each isolate exposed to subinhibitory concentrations of Penicillin G. * indicates the significant values (*p* < 0.05), reported in Table 1. The % of the dilutions are shown on the X axes, and the OD values are shown on the Y axes.

In contrast, Penicillin G allowed a significant increase (*p* < 0.05) of biofilm formation in four clinical isolates and one control isolate. Specifically, two isolates started to show a significantly increased biofilm formation in the presence of different subinhibitory concentrations (from 50% to 3.12% of MIC) (Figure 6).

The increase in biofilm formation was detected at a range of 1:2 to 1:64 of the MIC of the respective antibiotic. The isolates showed an enhancement in biofilm formation of up to 50%, and for some isolates, the values were doubled.

ANOVA tests on the differences between the subinhibitory concentrations and differences between the tested antibiotics revealed that in five isolates 1:2 MIC resulted in significant increases of OD, in two isolates 1:4 MIC resulted in significant increases of OD and in one isolate 1:8 MIC resulted in significant increases of OD (Table 2).

## 4. Discussion

### 4.1. Subinhibitory Concentrations of Different Antibiotics Increase Biofilm Production in E. faecalis

Antibiotics have provided the main solution to allow the human species to survive infections resulting from infectious diseases caused by bacteria that used to be lethal in the past [21]. However, the phenomenon of antibiotic resistance presented a new challenge for scientists who investigated the possible causes in several species. One of these areas of investigation is the aspect of subinhibitory concentrations. 

Jin et al. observed how *S. aureus* significantly increased biofilm formation when exposed to subinhibitory concentrations of Mupirocin [17]. Nagasawa et al. found that subinhibitory concentrations induce cell envelope stress phenomena in *S. mutans* [18]. 

*E. faecalis* is a human commensal that can cause severe nosocomial infections and is also associated with dental diseases, especially endodontic infections. Many isolates of this species are generally capable of biofilm formation [33]. Since there is evidence that subinhibitory antibiotic concentrations increase biofilm formation in certain bacterial species, this study investigated the effect of subinhibitory concentrations of different antibiotics on *E. faecalis* isolates in this regard, using microtiter-plates biofilm quantification technique. Biofilm assessments commonly rely on microtiter plate methodology, and in particular the crystal violet assay has recently been found to perform with a high repeatability and reproducibility [34]. The performed microtiter plate assay has been widely used for the characterization of microbial biofilm formation [28,29,35], however, supplementary tests to study the influence of sub-inhibitory antibiotic concentrations on biofilm formation of *E. faecalis* isolates should be added. Such tests should include i.e. a quantification of biofilm formation using confocal laser scanning microscopy (CLSM) and fluorescent dyes. Additionally, the influence of sub-inhibitory antibiotic concentrations on the expression of key genes involved in *E. faecalis* biofilm regulation could be analyzed. The most notable result was the ability of all six tested antibiotics to increase biofilm formation in the tested isolates at different subinhibitory concentrations. Vice versa, all of the tested isolates were influenced regarding their biofilm formation by at least one of the tested antibiotics.

As far as other bacterial species are concerned, the available literature on the effects of the subinhibitory concentrations of antibiotics is continuously increasing [36,37]. In fact, Waak et al. investigated the biofilm formation by means of the evaluation of OD, the exposure of *Streptococcus suis* to subinhibitory concentrations of Amoxicillin, Oxytetracycline and Lincomycin, and found that the sub-MIC increased the biofilm formation [37]. 

The topic of the subinhibitory concentrations of antibiotics affects not only the medical area, due to the abuse of antibiotics and the inappropriate prescriptions to patients, but also the food industry, due to the unsuitable use of these compounds in livestock farming. In fact, different strains of the same species that are isolated in different geographical areas can present different virulence factors such as antibiotic resistance or a different genotypical and phenotypical reaction to the subinhibitory concentrations of microbicidal molecules [38]. In particular, one isolate showed an increase of biofilm growth higher when exposed to subinhibitory concentrations of antibiotics than when non exposed. Microorganisms exposed to antibiotics show several stress responses, and can increase biofilm formation, dependent on the nature of the antibiotic, the dosage and the bacterial species or isolate [39]. The direct correlation between biofilm formation and antibiotic resistance however is not fully clarified [40]. 

### 4.2. Differences between Cell Wall Synthesis and Protein Synthesis Inhibitors

In this study, six isolates of *E. faecalis* from clinical endodontic infections and two control isolates were investigated to assess the effect of subinhibitory concentrations of several antibiotics on their capacity for biofilm formation. 

The isolates in the current study formed biofilm in the presence of sublethal concen-trations of antibiotics, a phenomenon that has already been reported for other bacterial taxa in the literature [16,17,33]. The antibiotics considered belong to different classes having two different modes of action: the inhibition of cell wall synthesis (Amoxicillin, Fosfomycin, Penicillin G and Vancomycin) and of protein synthesis (Doxycycline and Tetracycline). 

Regarding the inhibitors of cell wall synthesis, two clinical isolates and one of the con-trol isolates significantly increased their capacity for forming biofilm at sub-MIC of Amoxicillin, five clinical isolates and the two control isolates significantly increased their capacity for forming biofilm at sub-MIC of Fosfomycin, four clinical isolates and one control isolate significantly increased their capacity for forming biofilm at sub-MIC of Penicillin G and four clinical isolates and two control isolates significantly increased their capacity for forming biofilm at sub-MIC of Vancomycin. 

These results are consistent with the data in literature. Yu et al. reported the increased biofilm formation of *E. faecalis* in the presence of subinhibitory concentrations of cell wall synthesis inhibitors, including Fosfomycin, which is likely due to the cell lysis and increased presence of eDNA and eRNA [41]. Indeed, the current hypothesis on the mechanism of the biofilm induction from subinhibitory concentrations of antibiotics relies on the stress caused by the sub-MIC, provoking changes in the genotype of the microorganisms, and the release of the contents of the dead sensitive microbial cells, which contribute to the biofilm formation [42]. In terms of the protein synthesis inhib-itors, five clinical isolates and one of the control isolates significantly increased their capacity for forming biofilm at sub-MIC of Tetracycline and five clinical isolates and both control isolates significantly increased their capacity for forming biofilm at sub-MIC of Doxycycline. These data are in contrast with the data in literature. In this case, if we look at the study of Yu et al., which also included the protein synthesis in-hibitors, such as Doxycycline, the biofilm formation was not promoted in the subin-hibitory concentrations [41]. Instead, Tong et al. found how adjunctive Nisin to MTAD (an intracanal irrigant composed of 3% Doxycycline, 4.5% Citric acid, and 0.5% Poly-sorbate 80 detergent) and the creation of an alkaline environment caused an increased susceptibility of *E. faecalis* at sub-MIC exposure [43]. However, *E. faecalis*, a Gram-positive bacterium commonly inhabiting the gastrointestinal tract of mammals, is a species that easily develops antibiotic resistance and tolerance, and one of the top five most frequently isolated opportunistic pathogens in adults with health care-associated infections [44]. 

The development of biofilm-associated antibiotic tolerance in *E. faecalis* is not yet fully understood, but multiple genes have been reported to be involved. Dalle et al. reported fsr quorum-sensing regulon (fsrA, fsrC, and gelE) and two glycosyltransferases (epaI and epaOX) as genes associated with the development of *E. faecalis* biofilm in the presence of antibiotics [45]. In particular, the tested antibiotics were Daptomycin and Linezolid [45]. Scornec et al. showed how sub-MIC of Tetracycline can cause the transfer of the transposon Tn916, which hypothetically can lead to strains becoming resistant to mi-crobicidal molecules when resistance genes are transferred along with the transposon [46].

The T-tests results looked at the individual sub-MICs of the different classes of antibi-otics, and the most critical concentration was the 1:2 MIC in both classes of antibiotics: the cell wall synthesis inhibitors significantly increased the OD values in five isolates and the protein synthesis inhibitors significantly increased the OD values in three iso-lates. These data, together with the data about 1:4 and 1:8 MIC, in which the most critical antibiotics were Fosfomycin and Vancomycin, seem to support how the lytic action of the cell wall on the weaker subpopulation of microorganisms induces and improves the capacity of biofilm formation in this type of strain, due to the release of those molecules that can act as a substratum for biofilm formation [41,45].

In the case of *E. faecalis*, the protein synthesis inhibitors are therefore a class of antibi-otics that is less affected by the side effects of sub-MIC in terms of biofilm formation. Particularly Tigecycline, a protein synthesis inhibitor, has been reported to have the “suppression” property. This molecule does not select microorganisms with resistance profiles and therefore is a powerful weapon in the antibiotic therapy spectrum [47]. 

### 4.3. E. faecalis from Endodontic Lesions and the Higher Risks of Sub-MIC Exposure

As reported by Ranieri et al., the mechanisms for the enhanced biofilm formation in microorganisms exposed to sub-MIC are still not fully understood and remain a topic of debate due to the rapid development of antibiotic resistance [48]. 

If the misuse of antibiotics has led to the development of antibiotic resistance, with serious implications in those patients with immune systems that are already compromised, the root canal treatment and the therapy of secondary periapical infections represents those cases where it can easily happen that the concentration of the antibiotics used do not reach the MIC [19,28,49,50].

In a recent survey by Abraham et al. on dentists and endodontists in United Arab Emirates, about 12% of the interviewed dentists inappropriately prescribed antibiotics in hypothetical cases of irreversible pulpitis and about 20% inappropriately prescribed antibiotics in hypothetical cases of necrosis of pulp tissue with no systemic involvement [51]. A similar Brazilian study revealed that 88% of interviewed dentists would prescribe antibiotics in the latter scenario [52]. A recent study by our group revealed a high bacterial diversity in endodontic infections of patients who recently received systematic antibiotic therapy [53]. This emphasizes the fact that no effective antibiotic concentration can be achieved in infected root canals during a systematic therapy with different antibiotics. 

*E. faecalis* is often found in endodontic secondary infections and can be the cause of serious infections in other parts of the body [20]; its behavior in response to sub-MIC represents a crucial factor for a sufficient dental and medical therapy. 

Significantly, due to the narrow anatomical space of the root canals [54,55], the disinfecting irrigants as well as the instruments might not be able to fully reach the periapical lesions at the optimal concentrations to be active in terms of microbicidal effects and mechanical biofilm removal. These factors may lead to the failure of endodontic therapy with a risk of focal disease development, the failure in the periapical bone regeneration and the need to proceed to surgical interventions, including apicectomy or tooth extraction and the placement of bone substitutes to restore the quantity of bone loss due to the permanence of the infection [22,23,24,28,50]. 

The data from our study show how the sub-MIC of Doxycycline, which is frequently used as topical antibiotic in endodontics, surprisingly led to an increased capacity for biofilm formation. The topical use of Doxycycline is indeed a strategy to avoid the systemic administration of antibiotics and is therefore used in periodontal diseases [12,13]. For this reason, the topical use of this antibiotic in endodontics has been explored and is still the object of investigation. In an attempt to prevent biofilm formation, a recent study by Arias-Moliz et al. showed how the functionalization of nanoparticle polymers with Doxycycline inhibited the biofilm formation of *E. faecalis* [56].

Also the data on the sub-MIC of Tetracycline represent an important topic of discussion, since tetracyclines such as demeclocycline and minocycline are present as one of the main ingredient of intracanal antibiotic pastes [57]. Indeed, as previously reported [58], the antimicrobial effect decreases in the periphery of the root. Conclusively, the data of our study emphasize the importance of a correct use of this antibiotic, both in endodontics and in periodontics. In cases of endodontic treatment, correct mechanical preparation of the root canals is fundamental to allow the antibiotic to reach every part of the dentinal surfaces. In cases of periodontic treatment, it is important to assure mechanical instrumentation and subgingival debridement [4]. These precautions would avoid the exposure of the microbial biofilm to sub-MIC and thus to further or increased biofilm formation. 

## 5. Conclusions

The exposure to sub-MIC constitutes one of the stress factors that influence the genotype and phenotype of microorganisms, increasing the resistance of endodontic *E. faecalis* by enhancing biofilm formation. A conscious and correct use of antibiotic compounds, together with the functionalization and the conditioning of the surfaces, are necessary to decrease the risk of resistant bacteria selection.

## Figures and Tables

**Table 1:** Increase of biofilm formation of *E. faecalis* isolates exposed to subinhibitory antibiotic concentrations. Antibiotic concentrations that significantly increased the OD values in biofilm measurement are shown.

*E. faecalis* Isolate Number	Amoxicillin	Doxycycline	Fosfomycin	Penicillin G	Tetracycline	Vancomycin
12sp	N.S. ^1^	1:2 MIC–50%; 1:4 MIC–25%; 1:8 MIC–12.5%; 1:16 MIC–6.25%; 1:32 MIC–3.125%; 1:64 MIC–1.56% ***p*** **< 0.05**	1:2 MIC–50%; 1:4 MIC–25%; 1:8 MIC–12.5%; 1:16 MIC–6.25%; ***p*** **< 0.05**	1:2 MIC–50%; 1:4 MIC–25%; 1:16 MIC–6.25%; ***p*** **< 0.05**	1:2 MIC–50%; 1:4 MIC–25%; 1:8 MIC–12.5%; 1:16 MIC–6.25%; 1:32 MIC–3.125%; 1:64 MIC–1.56% ***p*** **< 0.05**	1:2 MIC–50%; ***p*** **< 0.05**
11sp	1:2 MIC–50%; 1:4 MIC–25%; 1:8 MIC–12.5%; ***p*** **< 0.05**	1:2 MIC–50%; ***p* < 0.05**	1:2 MIC–50%; 1:4 MIC–25%; 1:8 MIC–12.5%; 1:16 MIC–6.25%; 1:32 MIC–3.125%; 1:64 MIC–1.56% ***p*** **< 0.05**	1:2 MIC–50%; 1:4 MIC–25%; ***p* < 0.05**	1:2 MIC–50%; 1:4 MIC–25%; 1:8 MIC–12.5%; 1:16 MIC–6.25%; 1:32 MIC–3.125%; ***p*** **< 0.05**	1:2 MIC–50%; 1:8 MIC–12.5%; ***p*** **< 0.05**
RGFR	1:2 MIC–50%; 1:4 MIC–25%; ***p* < 0.05**	1:2 MIC–50%; 1:4 MIC–25%; ***p* < 0.05**	1:2 MIC–50%; 1:4 MIC–25%; 1:8 MIC–12.5%; 1:16 MIC–6.25%; 1:32 MIC–3.125%; 1:64 MIC–1.56% ***p*** **< 0.05**	1:2 MIC–50%; 1:8 MIC–12.5%; ***p*** **< 0.05**	1:2 MIC–50%; 1:8 MIC–12.5%; 1:16 MIC–6.25%; ***p*** **< 0.05**	1:2 MIC–50%; 1:4 MIC–25%; ***p*** **< 0.05**
1R8	N.S. ^1^	1:2 MIC–50%; 1:4 MIC–25%; 1:8 MIC–12.5%; 1:16 MIC–6.25%; 1:32 MIC–3.125%; 1:64 MIC–1.56% ***p* < 0.05**	1:2 MIC–50%; 1:4 MIC–25%; ***p*** **< 0.05**	1:2 MIC–50%; 1:4 MIC–25%; 1:8 MIC–12.5%; ***p*** **< 0.05**	1:2 MIC–50%; 1:4 MIC–25%; 1:8 MIC–12.5%; 1:16 MIC–6.25%; 1:32 MIC–3.125%; 1:64 MIC–1.56% ***p*** **< 0.05**	1:2 MIC–50%; ***p*** **< 0.05**
1R1	N.S. ^1^	N.S. ^1^	1:8 MIC–12.5%; 1:16 MIC–6.25%; 1:32 MIC–3.125%; ***p*** **< 0.05**	N.S. ^1^	N.S. ^1^	N.S. ^1^
21sp	N.S. ^1^	1:2 MIC–50%; 1:4 MIC–25%; 1:8 MIC–12.5%; 1:16 MIC–6.25%; 1:32 MIC–3.125%; 1:64 MIC–1.56% ***p*** **< 0.05**	N.S. ^1^	N.S. ^1^	1:8 MIC–12.5%; ***p*<0.05**	1:2 MIC–50%; ***p*** **< 0.05**
T9 ^2^	N.S. ^1^	1:4 MIC–25%; 1:8 MIC–12.5%; ***p* < 0.05**	1:2 MIC–50%; 1:8 MIC–12.5%; ***p*** **< 0.05**	N.S. ^1^	1:4 MIC–25%; 1:16 MIC–6.25%; ***p*** **< 0.05**	1:2 MIC–50%; 1:8 MIC–12.5%; ***p*** **< 0.05**
ATCC–29212	1:8 MIC–12.5% ***p*** **< 0.05**	1:4 MIC–25%; 1:8 MIC–12.5%; ***p* < 0.05**	1:2 MIC–50%; 1:4 MIC–25%; 1:8 MIC–12.5%; 1:16 MIC–6.25%; ***p*** **< 0.05**	1:4 MIC–25%; 1:8 MIC–12.5%; 1:16 MIC–6.25%; 1:32 MIC–3.125%; ***p*** **< 0.05**	N.S. ^1^	1:2 MIC–50%; 1:4 MIC–25%; 1:8 MIC–12.5%; ***p* < 0.05**

^1^ Not significant. ^2^ Laboratory control strain, high biofilm producer.

**Table 2:** Summary of T-test pairwise comparisons results displaying antibiotics with significant (*p* < 0.05) differences at the considered sub-MIC and isolates. Significant differences between antibiotics within different MIC-subgroups are marked with different letters (a, b, c, d). Only dilutions smaller than 1 (i.e. from 1/2) were considered. At 1/16, there were no more values for all antibiotics that made a comparison meaningful.

Isolate	1:2 MIC	1:4 MIC	1:8 MIC
11sp	Doxycycline ^a,c,d^ Tetracycline ^b,d^ Fosfomycin ^a,b,c^ Vancomycin ^b,d^	N.S. ^1^	N.S. ^1^
RGFR	Doxycycline ^b,c^ Amoxicillin ^a,c,d^ Fosfomycin ^a,b,d^ Penicillin G ^a,b,d^	Fosfomycin ^a^ Amoxicillin ^b^ Penicillin G ^b^ Vancomycin ^b^	N.S. ^1^
1R1	Doxycycline ^a^ Fosfomycin ^b^ Vancomycin ^c^	Doxycycline ^a^ Fosfomycin ^b^ Vancomycin ^c^	Fosfomycin ^a^ Vancomycin ^b^
21sp	Tetracycline ^b^ Fosfomycin ^a^ Vancomycin^c^	N.S. ^1^	N.S. ^1^
ATCC-29212	Tetracycline ^b^ Amoxicillin ^a^	N.S. ^1^	N.S. ^1^

^1^ Not Significant.

## Data Availability

The datasets used or analyzed during this study are available from the corresponding author on reasonable request.
